# Prognostic significance of IL-6 and IL-8 ascites levels in ovarian cancer patients

**DOI:** 10.1186/1471-2407-11-210

**Published:** 2011-05-30

**Authors:** Denis Lane, Isabelle Matte, Claudine Rancourt, Alain Piché

**Affiliations:** 1 Département de Microbiologie et Infectiologie, Faculté de Médecine, Université de Sherbrooke, 3001, 12ième Avenue Nord, Sherbrooke, J1H 5N4, Canada

## Abstract

**Background:**

The acellular fraction of epithelial ovarian cancer (EOC) ascites promotes *de novo *resistance of tumor cells and thus supports the idea that tumor cells may survive in the surrounding protective microenvironment contributing to disease recurrence. Levels of the pro-inflammatory cytokines IL-6 and IL-8 are elevated in EOC ascites suggesting that they could play a role in tumor progression.

**Methods:**

We measured IL-6 and IL-8 levels in the ascites of 39 patients with newly diagnosed EOC. Commercially available enzyme-linked immunosorbent assay (ELISA) was used to determine IL-6 and IL-8 ascites levels. Ascites cytokine levels were correlated with clinicopathological parameters and progression-free survival.

**Results:**

Mean ascites levels for IL-6 and IL-8 were 6419 pg/ml (SEM: 1409 pg/ml) and 1408 pg/ml (SEM: 437 pg/ml) respectively. The levels of IL-6 and IL-8 in ascites were significantly lower in patients that have received prior chemotherapy before the surgery (Mann-Whitney U test, *P *= 0.037 for IL-6 and *P *= 0.008 for IL-8). Univariate analysis revealed that high IL-6 ascites levels (*P *= 0.021), serum CA125 levels (*P *= 0.04) and stage IV (*P *= 0.009) were significantly correlated with shorter progression-free survival. Including these variables in a multivariate analysis revealed that elevated IL-6 levels (*P *= 0.033) was an independent predictor of shorter progression-free survival.

**Conclusion:**

Elevated IL-6, but not IL-8, ascites level is an independent predictor of shorter progression-free survival.

## Background

The incidence of ascites in women presenting with epithelial ovarian cancer (EOC) ranges from 45% to 75% depending on the tumor type but increases in advanced stages [1]. It is a distressing complication carrying substantial morbidity [2]. Unlike most stroma surrounding solid tumors, ascites constitute a unique form of tumor microenvironment. Recent evidences suggest than ascites play an active role in tumor development. EOC ascites may affect cell behaviour such as cell growth, invasion, and *de novo *drug resistance of EOC cells [3-5]. We recently reported that the acellular fraction of EOC ascites inhibits drugs- and death receptor-induced apoptosis *in vitro *(*de novo *resistance) [3,4]. Newly diagnosed women with protective ascites had significantly shorter progression-free survival [6] suggesting that ascites-mediated *de novo *resistance impacts on EOC progression. Stromal myofibroblasts and endothelial cells, adjacent to cancer cells in solid tumors, are replaced by floating mesothelial cells and by a variety of immune cells in ascites. The acellular fraction of ascites is a complex exudative fluid known to contain growth factors [7-9], lysophosphatidic acid (LPA) [10,11], cytokines [12,13] and extracellular matrix constituents (ECM) [14]. The contribution of these molecules in ovarian cancer progression is not well defined.

Although a wide variety of cytokines can be measured in ovarian cancer ascites, interleukin-6 (IL-6) and interleukin-8 (IL-8) are among the most abundant [12]. The concentration of these pro-inflammatory cytokines in ascites is 40- to 500-fold higher as compared to the levels found in serum [12]. IL-6 can be secreted in ascites by ovarian cancer cells, tumor-associated macrophages and peritoneal mesothelial cells. However, levels of IL-6 secreted by mesothelial cells are 600-fold higher than those secreted by ovarian cancer cells [15]. The source of the IL-8 found in ascites has not been well defined. These pro-inflammatory cytokines are involved in different pathophysiological processes including carcinogenesis. In ovarian cancer, IL-6 is thought to be involved in host immune responses to the disease [16-18]. IL-6 has also been demonstrated to be involved in autocrine growth of ovarian cancer cells [19-21]. IL-6 signaling in ovarian cancer cells can regulate tumor cell proliferation, invasion and angiogenesis [22-24] IL-8 was recently reported to promote ovarian tumor growth *in vivo *[25]. Despite these data, the biological relevance of high levels of IL-6 and IL-8 in ovarian cancer ascites remains mostly unknown. A number of studies have reported an association between serum levels of IL-6 and prognosis, and elevated levels correlated with a poor relapse-free and overall survival [26,27]. However, others have not found such correlation between elevated serum levels of IL-6 and survival time [28].

Based on the observation that ovarian cancer ascites may affect tumor progression and reported elevated levels of IL-6 and IL-8 in ascites, we hypothesize that these cytokines might affect the clinical progression of patients with ovarian cancer. The purpose of the present study was to investigate the prognostic significance of IL-6 and IL-8 ascites levels on progression-free survival in a cohort of 39 ovarian cancer patients.

## Methods

### Patients

The study population consisted of 39 patients with newly diagnosed epithelial ovarian cancer admitted at the Centre Hospitalier Universitaire de Sherbrooke. Informed consent was obtained from women that undergone surgery by the gynecologic oncology service between 2000 and 2009 for this institutional review board approved protocol. Baseline characteristics and serum CA125 levels were collected for all patients. All patients had a follow up > 1 year. Disease progression was defined by either CA125 ≥ 2 × nadir value on two occasions, documentation of increase or new lesions on CT-scan or death [29]. Patient's conditions were staged according to the criteria of the International Federation of Gynecology and Obstetrics (FIGO).

### Ascitic fluids

Peritoneal fluids were obtained at the time of initial cytoreductive surgery for all patients. Peritoneal fluids were centrifuged at 1000 rpm for 15 min and supernatants were stored at -80°C until assayed. All fluids were supplied by the Banque de tissus et de données of the Réseau de Recherche sur le Cancer des Fonds de la Recherche en Santé du Québec affiliated to the Canadian Tumor Repository Network (CTRNet).

### Determination of IL-6 and IL-8 concentration

Determination of IL-6 and IL-8 concentration was performed using the commercially available Quantikine kits from R&D Systems (Minneapolis, MN). The detection thresholds were 0.79 pg/ml for IL-6 and 3.5 pg/ml for IL-8. The intra-assay variability was for IL-6 and IL-8 analysis was 5-10% and 5-20% respectively. All tests were run in duplicates according to the manufacturer's instructions.

### Statistical analysis

Comparison between unpaired groups was made using the Mann-Whitney test or the Kruskal-Wallis test. The Kaplan-Meier method was used for the progression-free survival and Cox regression analysis and log-rank test were used for the statistical analysis. Progression-free survival was defined as the interval between the surgery and the time of disease progression. Univariate and multivariate analyses were performed using Cox regression. The threshold for statistical significance is a probability of 0.05.

## Results

A group of 39 patients with newly diagnosed epithelial ovarian cancer and for which ascites was available was evaluated. The mean concentration of IL-6 and IL-8 was determined by ELISA in the 39 ascites samples. The patient's clinicopathological characteristics are summarized in Table 1 according the IL-6 and IL-8 ascites levels. Mean ascites levels for IL-6 and IL-8 were 6419 pg/ml (SEM: 1409 pg/ml) and 1408 pg/ml (SEM: 437 pg/ml) respectively. We found no significant correlation between IL-6 or IL-8 ascites levels and FIGO stage, histopathology, grade, serum CA125 levels or the presence of post-operative residual tumor (> 2 cm). However, the levels of IL-6 and IL-8 in ascites were significantly lower in patients that have received prior chemotherapy before the surgery (Mann-Whitney U test, *P *= 0.037 for IL-6 and *P *= 0.008 for IL-8). It should be noted that the number of patients with post-operative residual tumor was limited (n = 4).

**Table 1:** IL-6 and IL-8 ascites levels in relation to different clinicopathological parameters

	No of ascites tested (%)	IL-6 mean pg/ml (SEM)	IL-8 mean pg/ml (SEM)
**Total**	39	6419 (1409)	1408 (437)
**Stage**			
I	9 (23)	4526 (3081)	1447 (997)
II	4 (10)	8893 (4719)	2738 (1786)
III	15 (39)	6550 (2521)	1181 (685)
IV	11 (28)	6514 (1869)	1119 (607)
**Histopathology**			
Serous	26 (67)	5209 (1484)	801 (339)
Mucinous	3 (8)	12641 (7227)	3375 (2596)
Mixed	7 (18)	8916 (4084)	2991 (1500)
Endometrioid	1 (2)	7896 (0)	463(0)
Other	2 (5)	1277 (583)	1268 (590)
**Grading**^a^			
1	4 (10)	3102 (1625)	228 (82)
2	10 (27)	8482 (3181)	842 (538)
3	20 (51)	5831 (1739)	1755 (661)
**Residual tumor**^a^			
≤ 2 cm	34 (87)	6286 (1487)	1555 (495)
> 2 cm	4 (10)	7457 (731)	378 (155)
**Prior chemotherapy**^a^			
Yes	4 (10)	444 (111)	430 (123)
No	34 (87)	6984 (1453)^b^	1972 (681)^b^
**Serum CA125**^a^			
< 319	19 (49)	4063 (1841)	1372 (806)
> 319	19 (49)	4406 (1765)	1491 (788)

^a ^Data unavailable for grade (n = 4), residual tumor (n = 1), prior chemotherpay (n = 1), serum CA125 (n = 1)

^b ^IL-6, *P *= 0.0001; IL-8, *P *= 0.002

**Figure 1:**
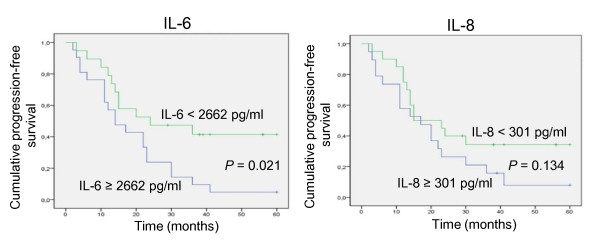
Kaplan-Meier analysis of progression-free survival in patients with or without elevated levels of IL-6 and IL-8 in ascites.

Progression-free survival analysis in the overall patient population showed a shorter progression-free survival for patients with a median IL-6 ascites levels > 2662 pg/ml as compared with those presenting with a median IL-6 levels < 2662 pg/ml (log rank test, *P *= 0.021) (Figure 1). Median progression-free survival was 14 months for patients with high IL-6 levels versus 24 months for those with low IL-6. A cutoff value of 2662 pg/ml was selected according to the median ascites levels in the 39 patients. Patients with high levels of IL-6 were 2.3 times (95% CI, 1.09 - 4.84) more likely to have disease progression as compared to those with low (< 2662 pg/ml) IL-6 ascites levels. High levels of IL-8 (median ≥ 301 pg/ml) were not significantly associated with increased risk of disease progression or shorter progression-free survival (Figure 1).

Univariate analysis showed that high IL-6 ascites levels (*P *= 0.021), serum CA125 levels (*P *= 0.04) and stage IV (*P *= 0.009) were significantly correlated with shorter progression-free survival (Table 2). Entering these variables in a multivariate Cox model analysis revealed that median IL-6 ascites levels > 2662 pg/ml was an independent predictor variable of shorter progression-free survival (*P *= 0.033). Although it did not quite reach statistical significance (*P *= 0.076), entering residual tumor > 2 cm in the multivariate model still showed that high IL-6 levels was significantly correlated with shorter progression-free survival.

**Table 2:** Univariate analysis of progression-free survival with selected factors

Variable	Median progression-free survival (months)	Univariate analysis (*P *value)
**IL-6 (pg/ml)**		
< 2662	24	
≥ 2662	14	0.021
		
**Stade**		
I	36	
IV	14	0.009
		
**Serum CA125 (U/ml)**		
< 319	29	
> 319	14	0.040
		
**Residual tumor**		
≤ 2 cm	20	
> 2 cm	11	0.076

## Discussion

The successful treatment of EOC remains a major challenge. Most (> 85%) EOC patients presenting with advanced disease will relapse. Recurrence defines incurable disease in most cases. The main obstacle to effective treatment is the failure of initial therapy to eradicate a sufficient number of tumor cells to prevent disease recurrence. One emerging model for the persistence of tumor cells after chemotherapy invokes ascites as a tumor microenvironment that promotes *de novo *drug resistance. Acellular fractions of EOC ascites have been shown to have anti-apoptotic activities [3,4] and ascites with pro-survival activities against cytotoxic agents were recently shown to significantly correlate with shorter progression-free survival in ovarian cancer patients [6]. These findings suggest that EOC ascites contain factors that affect progression-free survival by promoting tumor cell survival. EOC ascites contain a variety of soluble factors, including cytokines, which may contribute to *de novo *resistance of tumor cells. Because of the high levels of IL-6 and IL-8 found in ascites and the prosurvival activity of ascites, we hypothesized that IL6 and IL-8 could impact on progression-free survival. The identification of biomarkers that predict tumor progression would contribute to stratify patients for the selection of initial therapy.

We measured IL-6 and IL-8 levels in ascites samples obtained at the time of the initial surgery in a group of 39 patients with previously untreated EOC. Mean ascites levels of IL-6 and Il-8 found in our cohort were comparable to those previously reported by Giuntoli *et al*. in 22 ascites samples from women with EOC [12]. Our results show that elevated IL-6, but not IL-8, levels in ascites of patients with EOC correlated with shorter progression-free survival. We found low IL-6 levels to correlate significantly with prior chemotherapy (*P *= 0.037). However, these data should be considered with caution given the small number of patients in the group that received chemotherapy prior to their surgery. No significant correlation was found between IL-6 and IL-8 levels in ascites and the other clinicopathological parameters such as stage, grade, histologic type and size of residual tumor left after initial debulking. These data are consistent with those of Tempfer *et al*. [26] and Scambia *et al*. [27] which showed that elevated IL-6 concentration in serum of ovarian cancer patients correlated with poor overall survival. They are also consistent with the observation that IL-6 promoter polymorphism, which may affect IL-6 levels, impacts on survival of women with ovarian cancer [30]. However, measuring IL-6 in ascites might actually be more relevant than measuring it in serum. IL-6 is released by the peritoneal mesothelial cells and concentrates in ascites [12,15] where it could affect tumor cell behaviour. IL-6 may be cleared rapidly from the circulation and consequently serum levels may not correlate with ascites levels.

Although, our data suggest that IL-6 levels in ascites are associated with shorter progression-free survival, the precise underlying molecular mechanisms responsible for these findings remain to be established. One possibility is that elevated ascites IL-6 levels promote *de novo *tumor cells resistance to chemotherapy contributing to earlier disease recurrence [31]. This is supported by the observation that IL-6 inhibited death receptor-induced cell death in long term cell viability assays (Lane, unpublished data). IL-6 signaling cascade in ovarian cancer cells has been associated with the development of Taxol resistance [32,33]. Alternatively, IL-6 could stimulate the proliferation of tumor cells leading to a shorter progression-free survival [18]. IL-6-mediated angiogenesis could also play a role [23]. Tumor angiogenesis plays an important role in cancer progression and metastasis. The angiogenesis and the disruption of vascular barrier both contribute to ascites formation [34]. Because IL-6 has been shown to be involved in tumor angiogenesis in ovarian cancer, IL-6 may be important in promoting the formation of ascites as well as the progression of ovarian cancer. IL-6 signalling prevents chemotherapy-induced endothelial cells apoptosis and the blockage of its signalling cascade was therapeutically beneficial in xenograft mouse model [35]. Thus, interference with IL-6 pathway may offer opportunities for ovarian cancer therapy. However, using a monoclonal antibody that specifically blocks IL-6 signaling, siltuximab, Guo *et al*. demonstrated that although the combination of siltuximab with paclitaxel increased the sensitivity of ovarian tumor cells to paclitaxel *in vitro*, the combination was ineffective *in vivo *in xenogrft mouse model [36]. Although clinical trials of monoclonal antibodies to IL-6 for the treatment of other types of cancer have shown encouraging results [37,38], a clear benefit for patients with ovarian cancer remains to be demonstrated. Finally, the high levels of IL-6 could enhance the immune suppressive status of the tumor microenvironment by inducing B7-H4 expression on tumor associated macrophages and promote apoptosis in these cells [39]. IL-6 may divert the immune response from Th1 towards a suppressive Th2 response although controversial data have been reported [11,40,41]. IL-6 may also contribute to reprogram the tumor adjacent stromal microenvironment which may facilitate dissemination to the peritoneal cavity. The contribution of this reactive stromal is being recognized as an important element for tumor progression [42].

## Conclusions

We found that high levels of IL-6 in ascites of newly diagnosed women with ovarian cancer significantly correlated with shorter progression-free survival. In contrast, ascites levels of IL-8 did not show such association. These data suggest that IL-6 could be an important component that contributes to ascites-mediated *de novo *drug resistance.

## Conflict of interest statement

The authors declare that they have no competing interests.

## Authors' contributions

DL participated in the design of the study and performed the assays for measuring IL-6 and IL-8 levels in ascites. IM was responsible for obtained the ascites and the clinical data. CR participated in the design of the study and helped to draft the manuscript. AP conceived the study, participated in its design and drafted the manuscript. All authors read and approved the final manuscript.

## Pre-publication history

The pre-publication history for this paper can be accessed here:

http://www.biomedcentral.com/1471-2407/11/210/prepub

## References

[B1] Partridge EE Barnes MN Epithelial ovarian cancer: prevention, diagnosis, and treatment CA Cancer J Clin 1999 49 297 320 10.3322/canjclin.49.5.297 11198956

[B2] Shen-Gunther J Mannel RS Ascites as a predictor of ovarian malignancy Gynecol Oncol 2002 87 77 83 10.1006/gyno.2002.6800 12468346

[B3] Lane D Robert V Grondin R Rancourt C Piché A Malignant ascites protect against TRAIL-induced apoptosis by activating the PI3K/Akt in human ovarian carcinoma cells Int J Cancer 2007 121 1227 1237 10.1002/ijc.22840 17534891

[B4] Lane D Goncharenko-Khaider N Rancourt C Piché A Ovarian cancer ascites protects from TRAIL-induced cell death through αvβ5 integrin-mediated focal adhesion kinase and Akt activation Oncogene 2010 29 3519 3531 10.1038/onc.2010.107 20400979

[B5] Puiffe ML Le Page C Filali-Mouhim A Zietarska M Ouellet V Tonin PN Chevrette M Provencher DM Mes-Masson AM Characterization of ovarian cancer ascites on cell invasion, proliferation, spheroid formation, and gene expression in an *in vitro *model of epithelial ovarian cancer Neoplasia 2007 9 820 829 10.1593/neo.07472 17971902 PMC2040209

[B6] Lane D Matte I Rancourt C Piché A The prosurvival activity of ascites against TRAIL is associated with a shorter disease-free interval in patients with ovarian cancer J Ovarian Res 2010 3 1 10.1186/1757-2215-3-1 20157422 PMC2821314

[B7] Mills GB May C McGill M Roifman CM Mellors A A putative new growth factor in ascitic fluid from ovarian cancer patients: identification, characterization, and mechanism of action Cancer Res 1988 48 1066 1071 3422589

[B8] Mills GB May C Hill M Campbell S Shaw P Marks A Ascitic fluid from human ovarian cancer patients contains growth factors necessary for intraperitoneal growth of human ovarian adenocarcinoma cells J Clin Invest 1990 86 851 855 10.1172/JCI114784 2394835 PMC296802

[B9] Richardson M Gunawan J Hatton MW Seidlitz E Hirte HW Singh G Malignant ascites fluid (MAF), including ovarian cancer-associated MAF, contains angiostatin and other factor(s) which inhibit angiogenesis Gynecol Oncol 2002 86 279 287 10.1006/gyno.2002.6760 12217749

[B10] Xu Y Gaudette DC Boynton JD Frankel A Fang XJ Sharma A Hurteau J Casey G Goodbody A Mellors A Holub BJ Mills GB Characterization of an ovarian cancer activating factor in ascites from ovarian cancer patients Clin Cancer Res 1995 1 1223 1232 9815916

[B11] Yamada T Sato K Komachi M Malchinkhuu E Tobo M Kimura T Kuwabara A Yanagita Y Ikeya T Tanahashi Y Ogawa T Ohwada S Morishita Y Ohta H Im DS Tamoto K Tomura H Okajima F Lysophosphatidic acid (LPA) in malignant ascites stimulates motility of human pancreatic cancer cells through LPA1 J Biol Chem 2004 279 6595 6605 14660630 10.1074/jbc.M308133200

[B12] Giuntoli RL Webb TJ Zoso A Rogers O Diaz-Montes TP Bristow RE Oelke M Ovarian cancer-associated ascites demonstrates altered immune environment: implications for antitumor immunity Anticancer Res 2009 29 2875 2884 19661290

[B13] Radke J Schmidt D Böhme M Schmidt U Weise W Morenz J Cytokine level in malignant ascites and peripheral blood of patients with advanced ovarian carcinoma Geburtshilfe Frauenheilkd 1996 56 83 87 10.1055/s-2007-1022247 8647364

[B14] Ahmed N Riley C Oliva K Rice G Quinn M Ascites induces modulation of α6β1 integrin and urokinase plasminogen activator receptor expression and associated functions in ovarian carcinoma Br J Cancer 2005 92 1475 1485 10.1038/sj.bjc.6602495 15798771 PMC2362012

[B15] Offner FA Obrist P Stadlmann S Feichtinger H Klinger P Herold M Zwierzina H Hittmair A Mikuz G Abendstein B Zeimet A Marth C Il-6 secretion by human peritoneal mesothelial and ovarian cancer cells Cytokine 1995 7 542 547 10.1006/cyto.1995.0073 8580370

[B16] Asschert JG Vellenga E Ruiters MH de Vries EG Regulation of spontaneous and TNF/IFN-induced IL-6 expression in two human ovarian-carcinoma cell lines Int J Cancer 1999 82 244 249 10.1002/(SICI)1097-0215(19990719)82:2<244::AID-IJC15>3.0.CO;2-N 10389759

[B17] Asschert JG de Vries EG De Jong S Withoff S Vellenga E Differential regulation of IL-6 promoter activity in a human ovarian-tumor cell line transfected with various p53 mutants: involvement of AP-1 Int J Cancer 1999 81 236 242 10.1002/(SICI)1097-0215(19990412)81:2<236::AID-IJC12>3.0.CO;2-R 10188725

[B18] Watson JM Sensintaffar JL Berek JS Martinez-Maza O Constitutive production of interleukin 6 by ovarian cancer cell lines and by primary ovarian tumor cultures Cancer Res 1990 50 6959 6965 2208162

[B19] Wu S Rodabaugh K Martinez-Maza O Watson JM Silberstein DS Boyer CM Peters WP Weinberg JB Berek JS Bast RC Stimulation of ovarian tumor cell proliferation with monocyte products inducing interleukin 1, interleukin 6 and tumor necrosis factor-alpha Am J Obstet Gynecol 1992 166 977 1007 10.1016/0002-9378(92)91379-o 1550178

[B20] Hsu SM Hsu PL Autocrine and paracrine functions of cytokines in malignant lymphomas Biomed Pharmacother 1994 48 433 444 10.1016/0753-3322(94)90004-3 7858153

[B21] Nakazaki H Preoperative and postoperative cytokines in patients with cancer Cancer 1992 70 709 713 10.1002/1097-0142(19920801)70:3<709::AID-CNCR2820700328>3.0.CO;2-O 1320454

[B22] Syed V Ulinski G Mok SC Ho SM Reproductive hormone-induced, STAT3-mediated interleukin 6 action in normal and malignant human ovarian surface epithelial cells J Natl Cancer Inst 2002 94 617 629 11959895 10.1093/jnci/94.8.617

[B23] Obata NH Tamakoshi K Shibata K Kikkawa F Tomoda Y Effects of interleukin-6 on in vitro cell attachment, migration and invasion of human ovarian carcinoma Anticancer Res 1997 17 337 342 9066674

[B24] Nilsson MB Langley RR Fidler IJ Interleukin-6, secreted by human ovarian carcinoma cells, is a potent proangiogenic cytokine Cancer Res 2005 65 10794 10800 10.1158/0008-5472.CAN-05-0623 16322225 PMC1534114

[B25] Shahzad MM Arevalo JM Armaiz-Pena GN Lu C Stone RL Moreno-Smith M Nishimura M Lee JW Jennings NB Bottsford-Miller J Vivas-Mejia P Lutgendorf SK Lopez-Berestein G Bar-Eli M Cole SW Sood AK Stress effects on FosB- and interleukin-8 (*IL8*)-driven ovarian cancer growth and metastasis J Biol Chem 2010 285 35462 35470 10.1074/jbc.M110.109579 20826776 PMC2975170

[B26] Tempfer C Zeisler H Sliutz G Haeusler G Hanzal E Kainz C Serum evaluation of interleukin 6 in ovarian cancer patients Gynecol Oncol 1997 66 27 30 10.1006/gyno.1997.4726 9234916

[B27] Scambia G Testa U Panici PB Foti E Martucci R Gadducci A Perillo A Facchini V Peschle C Mancuso S Prognostic significance of interleukin 6 serum levels in patients with ovarian cancer Br J Cancer 1995 71 354 356 10.1038/bjc.1995.71 7841052 PMC2033591

[B28] Plante M Rubin SC Wong GY Federici MG Finstad CL Gastl GA Interleukin-6 level in serum and ascites as a prognostic factor in patients with epithelial ovarian cancer Cancer 1994 73 1882 1888 10.1002/1097-0142(19940401)73:7<1882::AID-CNCR2820730718>3.0.CO;2-R 8137215

[B29] Rustin GJ Timmers P Nelstrop A Shreeves G Bentzen SM Baron B Piccart MJ Bertelsen K Stuart G Cassidy J Eisenhauer E Comparison of CA-125 and standard definitions of progression of ovarian cancer in the intergroup trial of cisplatin and paclitaxel versus cisplatin and cyclophosphamide J Clin Oncol 2006 24 45 51 10.1200/JCO.2005.01.2757 16382112

[B30] Garg R Wollan M Galic V Garcia R Goff BA Gray HJ Swisher E Common polymorphism in interleukin 6 influences survival of women with ovarian and peritoneal carcinoma Gynecol Oncol 2006 103 793 796 10.1016/j.ygyno.2006.08.043 17023036 PMC2562602

[B31] Johnson MT Gotlieb WH Rabbi M Martinez-Maza O Berek JS Induction of cisplatin resistance and metallothionein expression by interleukin-6 Gynecol Oncol 1993 49 110

[B32] Duan Z Foster R Bell DA Mahoney J Wolak K Valdya A Hampel C Lee H Seiden MV Signal transducers and activators of transcription 3 pathway activation in drug-resistant ovarian cancer Clin Cancer Res 2006 12 5055 5063 10.1158/1078-0432.CCR-06-0861 16951221

[B33] Duan Z Feller AJ Penson RT Chabner BA Seiden MV Discovery of differentially expressed genes associated with paclitaxel resistance using cDNA array technology: analysis of interleukin (IL)-6, IL-8, and monocyte chemotactic protein 1 in the paclitaxel-resistance phenotype Clin Cancer Res 1999 5 3445 3453 10589757

[B34] Mesiano S Ferrara N Jaffe RB Role of vascular endothelial growth factor in ovarian cancer: inhibition of ascites formation by immunoneutralization Am J Pathol 1998 153 1249 1256 10.1016/S0002-9440(10)65669-6 9777956 PMC1853065

[B35] Lo CW Chen MW Hsiao M Wang S Chen CA Hsiao SM Chang JS Lai TC Rose-John S Kuo ML Wei LH IL-6 trans-signaling in formation and progression of malignant ascites in ovarian cancer Cancer Res 2011 71 424 434 10.1158/0008-5472.CAN-10-1496 21123455

[B36] Guo Y Nemeth J O'Brien C Susa M Liu X Zhang Z Choy E Mankin H Hornicek F Duan Z Effects of siltuximab on the IL-6-induced signaling pathway in ovarian cancer Clin Cancer Res 2010 16 5759 5769 10.1158/1078-0432.CCR-10-1095 20699329

[B37] Van Rhee F Fayad L Voorhees P Furman R Lional S Borghaei H Sokol L Crawford J Cornfeld M Qi M Qin X Herring J Casper C Kurzrock R Siltuximab, a novel anti-interleukin-6 monoclonal antibody, for Castelman's disease J Clin Oncol 2010 28 3701 3708 10.1200/JCO.2009.27.2377 20625121

[B38] Rossi JF Fegueux N Lu ZY Legouffe E Exbrayat C Bozonnat MC Navarro R Lopez E Quittet P Daures JP Rouille V Kanouni T Widjenes J Klein B Optimizing the use of anti-interleukin-6 monoclonal antibody with dexamethasone and 140 mg/m2 of melphalan in multiple myeloma: results of a pilot study including biological aspects Bone Marrow Transplantation 2005 36 771 779 10.1038/sj.bmt.1705138 16113665 PMC2034604

[B39] Kryczek I Zou L Rodriguez P Zhu G Wei S Mottram P Brumlik M Cheng P Curiel T Myers L Lackner A Alvarez X Ochoa A Chen L Zou W B7-H4 expression identifies a novel suppressive macrophage population in human ovarian carcinoma J Exp Med 2006 203 871 881 10.1084/jem.20050930 16606666 PMC2118300

[B40] Punnonen R Teisala K Kuoppala T Bennett B Punnonen J Cytokine production profiles in the peritoneal fluids of patients with malignant or benign gynecologic tumors Cancer 1998 83 788 796 10.1002/(SICI)1097-0142(19980815)83:4<788::AID-CNCR24>3.0.CO;2-N 9708947

[B41] Yigit R Figdor CG Zusterzeel PM Pots JM Torensma T Massuger LG Cytokine analysis as a tool to understand tumour-host interaction in ovarian cancer Eur J Cancer 2011 10.1016/j.ejca.2011.03.026 21514148

[B42] Schauer IG Sood AK Mok S Liu J Cancer-associated fibroblasts and their putative role in potentiating the initiation and development of epithelial ovarian cancer Neoplasia 2011 13 393 405 21532880 10.1593/neo.101720 PMC3084616

